# Urethral Polyembolokoilamania: An Unusual Manifestation of Behavioral and Psychological Symptoms of Dementia (BPSD)

**DOI:** 10.1155/2018/3018378

**Published:** 2018-11-22

**Authors:** Reem M. A. Shafi, Laura Suarez, Maria I. Lapid

**Affiliations:** Mayo Clinic, Department of Psychiatry and Psychology, Rochester, Minnesota, USA

## Abstract

Behavioral and psychological symptoms of dementia (BPSD) have varied presentations and frequently occur throughout the trajectory of dementia. Hypersexuality and general disinhibition of societal and cultural norms are commonly documented in all types of dementia. However, sparse literature exists on polyembolokoilamania (insertion of foreign objects in bodily orifices) without a sexual component as a dementia-related symptom. We review an unusual case of a 94-year-old man who presented with urethral polyembolokoilamania without hypersexuality or other behavioral disinhibition. We highlight clinical considerations of managing urethral polyembolokoilamania in an elderly patient without a previous neurocognitive disorder diagnosis. A multidisciplinary team approach with input from Internal Medicine, Urology, Psychiatry, and Neurology lead to a comprehensive assessment of a patient that could have been managed solely as a surgical case. This spearheaded a formal diagnosis of neurocognitive disorder—guiding successful management, follow-up, caregiver education, and reduction of further harm.

## 1. Introduction

Behavioral and psychological symptoms of dementia (BPSD) can occur at any stage in the illness trajectory. They are widely grouped into categories of pathologically dysfunctional or altered mood, emotions, thought, perception, motor activity, and personality traits [1]. Overall up to 97% of patients with dementia have a BPSD symptom [2–5]. BPSD contribute to poor quality of life, burdensome healthcare costs, and the institutionalization of people with dementia. BPSD are not currently incorporated in diagnostic criteria for neurocognitive disorder [1].

Polyembolokoilamania is a Greek derivative describing the behavioral phenomenon of the insertion of foreign bodies into bodily orifices [6]. Accidental insertion can be common in young children and is a particular trait of Smith Magenis syndrome [7, 8]. It may reflect risk taking behavior, sexual experimentation or drug trafficking. Psychiatrically, it is a symptom manifestation of conditions including eating disorders, substance use disorders, psychosis, and factitious disorder [9]. Although sexual disinhibition is a common BPSD symptom, limited literature exists on polyembolokoilamania as a dementia-related symptom [9, 10]. Case reports detailing polyembolokoilamania in adults focus on autoerotic mishaps leading to medical and surgical complications including death [11–13].

We present a case of an elderly male referred to our consult liaison psychiatry service with urethral polyembolokoilamania. We highlight clinical considerations and how a multidisciplinary team approach spearheaded a neurocognitive disorder diagnosis resulting in successful management and caregiver education.

## 2. Case Presentation

### 2.1. Initial Presentation

94-year-old Caucasian male presented to his primary care provider with complaints of a “lump” in his scrotum with dysuria and incontinence. His past medical history included hypothyroidism, urinary retention with intermittent catheterization, and controlled atrial fibrillation. A pelvic ultrasound scan showed a perineal mass and he was urgently referred to the Emergency Room. Routine lab work including complete blood count, electrolytes, renal function, and international normalized ratio (INR) were unremarkable. His urine gram stain was negative.

**Figure 1:**
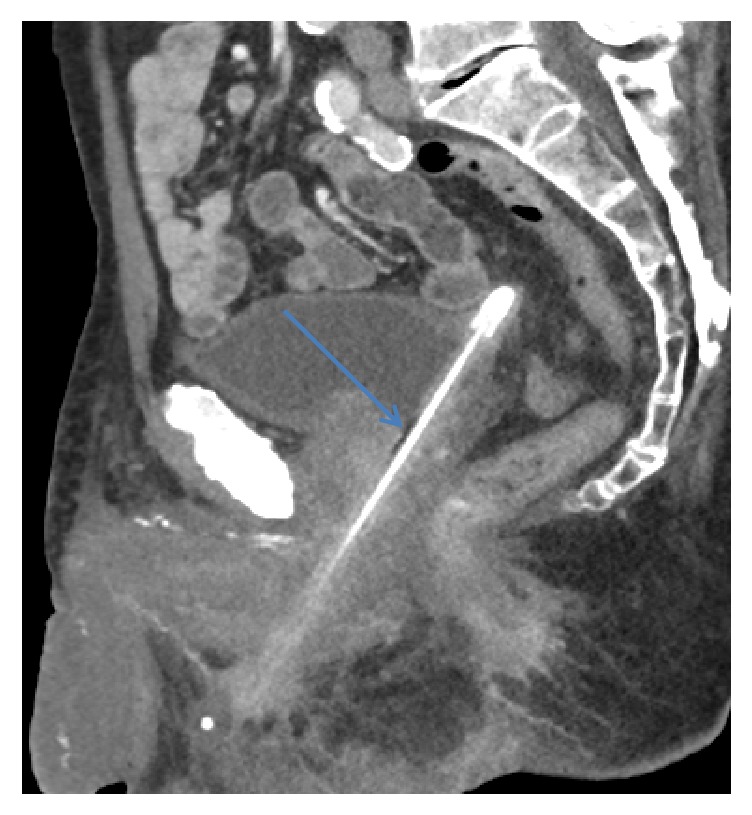
Sagittal images from CT pelvis showing pencil extending from the bladder dome (which is tented superiorly) through the prostatic urethra then through a perforation in the bulbar urethra.

**Figure 2:**
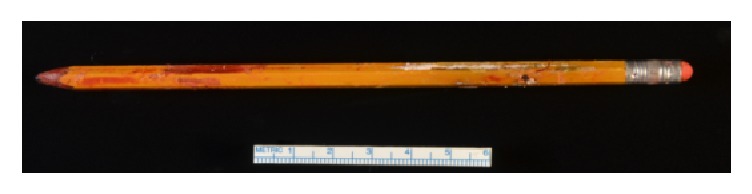
The pencil cystoscopically extracted in the operating room.

A pelvic computerized tomography (CT) scan showed a 16 cm foreign body within the bladder perforating the ventral surface of the bulbar urethra and extending into the perineal soft tissues (Figure 1). He was admitted to Medicine for anticoagulation reversal prior to cystoscopic removal of the specimen by Urology (Figure 2). An indwelling suprapubic catheter was subsequently placed with a plan to continue catheter placement upon discharge.

### 2.2. Psychiatric Assessment

He was referred to the psychiatry consult liaison service and evaluated pre- and postoperatively. There was no evidence of suicidality or a psychotic, mood, or delirious process. However his thought form was illogical and he denied knowledge of urethral placement of the object. Montreal Cognitive Assessment (MoCA) score was 15/30 [14].

His daughter (caregiver and guardian) described him as a retired mechanic who liked to “fix things”, was “very private”, and did not disclose symptoms readily. Previous episodes of urethral foreign body insertions (usually straws) had occurred when he attempted to self-manage urinary symptoms leading to urinary tract infections and abscesses.

He was dependent on basic activities of daily living (ADL) such as showering and instrumental ADL such as driving and managing financial transactions and had been a victim of a financial exploitation on the Internet. He had no previous evaluations for cognitive impairment and has no known disinhibition or hypersexuality.

The preliminary diagnostic impression was that of a major neurocognitive disorder. We outlined recommendations for laboratory tests, safety evaluation, imaging, and neurology consultation. The occupational and physical therapist assessment recommended continuous supervision. A dementia workup including folate, vitamin B12, human immunodeficiency virus (HIV) and thyroid function were unremarkable. Head CT showed generalized cerebral volume loss, leukoaraiosis, and hippocampal sclerosis. Neuropsychological testing indicated impairment in memory and executive function domains. A Neurology consultation confirmed a diagnosis of major neurocognitive disorder of mixed aetiology.

We did not recommend psychotropics since there was no mood, psychotic or other behavioral concerns. Nonpharmacological measures are the first line approach in managing BPSD symptoms [4]. Increased supervision and the placement of a suprapubic catheter reassured him that he was emptying his bladder fully. Successful management of his urinary symptoms has led to no further recurrences of urethral polyembolokoilamania.

## 3. Discussion

Due to the varied manifestations of BSPD and the lack of a core, unified definition these symptoms can be difficult to treat or identify. In the outlined case, the previous incidents of urethral polyembolokoilamania did not precipitate in a comprehensive cognitive assessment. This may also have been contributed to by his seemingly appropriate level of functioning at home.

A multidisciplinary team approach was essential in providing high level clinical care. The combination of Internal Medicine, Urology, Psychiatry, and Neurology meant that a holistic patient assessment was obtained, rather than approached as an isolated surgical presentation.

Delirium/encephalopathy was considered. However his lab work and longitudinal assessment by multiple specialists and multidisciplinary team members did not support this as the cause of the polyembolokoilamania.

The importance of reaching a formal diagnosis is highlighted here as it led to caregiver education, discussion of prognosis, and advanced care planning. The safety assessment was particularly important in light of his discharge to his daughter's home who did not wish to pursue institutionalization. Another important consideration is that dementia is a significant risk factor for elder abuse. Clinicians are encouraged to conduct thorough clinical assessments before disregarding elder abuse as a possibility in clinical presentations involving physical harm [15–17]. His low MoCA score (normal ≥26), inability to relay on a coherent narrative, and his initial denial of inserting the pencil meant we considered the risk of abuse [14–19]. Nutritional status, injuries, caregiver interview, and psychiatric assessment indicated a low clinical concern for elder abuse [17]. After rapport was established, he eventually disclosed self-insertion of the pencil.

Due to the heterogeneity of BPSD, clinicians should have a high index of suspicion for neurocognitive disorders in the elderly presenting with unusual behaviors such as polyembolokoilamania. A cognitive evaluation to rule out dementia is essential for appropriate management. A psychiatric consultation should be strongly considered as part of a holistic approach to identify psychiatric or neuropsychiatric disorders. Even if a psychiatric disorder is not found, harm reduction strategies and nonpharmacological interventions can be implemented to prevent harmful behavior [7].

## Abbreviations

BPSD: Behavioral and psychological symptoms of dementia
CT: Computerized tomography
INR: International Normalized Ratio
MoCA: Montreal Cognitive Assessment (MoCA).
